# An *in vitro* investigation of the apoptosis-inducing activity of corosolic acid in breast cancer cells

**DOI:** 10.22038/IJBMS.2023.67783.14834

**Published:** 2023-04

**Authors:** Saade Abdalkareem Jasim, Omer Zedan Khalaf, Shadia Hamoud Alshahrani, Kadda Hachem, Shukhrat Ziyadullaev, Abduladheem Turki Jalil, ChangMing Wang, Rahman S. Zabibah, Yousef A Bin Jardan, Qutaiba A. Qasim, Marwah Suliman Maashi, Yasser Fakri Mustafa

**Affiliations:** 1 Medical Laboratory Techniques Department, Al-Maarif University College, Al-anbar-Ramadi, Iraq; 2 Al-Anbar Health Directorate, Iraq; 3 Medical Surgical Nursing Department, King Khalid University, Almahala, Abha, Saudi Arabia; 4 Laboratory of Biotoxicology, Pharmacognosy and Biological Valorization of Plants (LBPVBP), Faculty of Sciences, University of Saida-Dr Moulay Tahar, 20000 Saida, Algeria; 5 Department of Internal Diseases, Vice-rector for Scientific Affairs and Innovations, Samarkand State Medical University, Amir Temur Street 18, Samarkand, Uzbekistan; 6 Medical Laboratories Techniques Department, Al-Mustaqbal University College, Babylon, Hilla, 51001, Iraq; 7 International College, Krirk University, Bangkok, 3 Ram Inthra Rd, Khwaeng Anusawari, Khet Bang Khen, Krung Thep Maha Nakhon, 10220, Thailand; 8 Medical Laboratory Technology Department, College of Medical Technology, Islamic University, Najaf, Iraq; 9 Department of Pharmaceutics, College of Pharmacy, King Saud University, Riyadh, Saudi Arabia; 10 College of Pharmacy, Al-Ayen University, Thi-Qar, Iraq; 11 Medical Laboratory Science Department, Faculty of Applied Medical Sciences, King Abdulaziz University, Jeddah 21589, Saudi Arabia; 12 Department of Pharmaceutical Chemistry, College of Pharmacy, University of Mosul, Mosul-41001, Iraq

**Keywords:** Apoptosis, Breast cancer, Corosolic acid, JAK2, STAT3

## Abstract

**Objective(s):**

Breast cancer is the most prevalent cancer among females with different molecular subtypes. Corosolic acid is a pentacyclic triterpenoid with anti-cancer properties.

**Materials and Methods:**

The MTT assay was used to assess the cytotoxic activity of corosolic acid on MDA-MB-231 and MCF7 cell lines. To determine the apoptotic cells, the flow cytometry technique was utilized. The expression levels of apoptosis-related genes and proteins were quantified using quantitative real time-PCR (qRT-PCR) and Western blotting methods. The activity of caspase enzymes was measured by spectrophotometry.

**Results:**

Corosolic acid significantly inhibited the proliferation of both cell lines compared with controls. This agent markedly induced apoptosis in MDA-MB-231 cells but did not affect MCF7 cells compared with controls. Treating the MADA-MB-231 and MCF7 cell lines with corosolic acid showed an inducing effect on apoptosis-associated caspases, including Caspase-8, 9, and -3, in MADA-MB-231 cells with no effect on apoptotic markers in MCF7 cells. Further experiments uncovered corosolic acid-induced apoptosis in MADA-MB-231 cells by decreasing the expression of the phosphorylated form of JAK2 and STAT3 proteins.

**Conclusion:**

The present data suggested that corosolic acid is an apoptosis-inducing phytochemical in triple-negative breast cancer MADA-MB-231 cells. Also, corosolic acid triggered apoptosis in these cells by stimulating both pathways of apoptosis and inhibiting the JAK/STAT signaling. Furthermore, corosolic acid was found to inhibit MCF7 cell proliferation by a non-apoptotic mechanism.

## Introduction

Breast cancer is the most prevalent type of cancer among females and ranks as the second cause of cancer-dependent death in women worldwide (1). Breast cancer has different molecular subtypes that are generally subcategorized based on the expression or lack of three cellular receptors, including estrogen receptor (ER), progesterone receptor (PR), and human epidermal growth factor receptor 2 (HER2) (2). Triple-negative breast cancer (TNBC) is a specific subtype of this cancer with no expression of ER, PR, and HER-2 (3). This subtype of breast cancer is highly metastatic with aggressive features and poor prognosis leading to unfavorable clinical outcomes (4). TNBC tumors don’t respond to endocrine therapy or HER2 treatment approaches due to the lack of receptors, and thus no standard treatment has been developed for TNBC (5). Therefore, the need to develop novel and effective therapeutic modalities for the treatment of TNBC has become an urgent clinical requirement.

Plant-derived natural products have been shown to act as new and effective anti-cancer agents (6, 7). Corosolic acid, also known as 2α-hydroxyursolic acid, is a pentacyclic triterpenoid found in *Lagerstroemia* *speciosa *with antiinflammatory, antiobesity, antidiabetic, antihyperlipidemic, anti-cancer, and antiviral activities (8). The leaves of *L. speciosa*, a tropical plant grown in different countries such as Malaysia, Vietnam, Philippines, China, Europe, and South America, are rich in corosolic acid (8). Corosolic acid has shown anti-tumor, anti-proliferative, apoptotic, and non-apoptotic activities against different human cancer cells by acting on various molecular pathways (9). This terpenoid significantly triggered apoptosis in the human colon cancer cell line, HCT116, by activating Caspase-8, -9, and -3 in these cancer cells (10). Corosolic acid suppresses the progression of hepatocellular carcinoma *in vitro* and hampers tumorigenesis in a xenograft model of this cancer *in vivo* (11). This compound also acts as an enhancer of some chemotherapy agents. For example, the combination of corosolic acid and 5Fluorouracil (5FU) more significantly blocked the mammalian target of rapamycin (mTOR) signaling pathway in SNU620 human gastric carcinoma cells and induced apoptosis in these cancer cells (12). 

Apoptosis is known as a process of programmed cell death in multicellular organisms and can be targeted as a strategy for the treatment of cancer (13). Aberrant alterations in the normal function of this process may result in abnormal cell growth and uncontrolled cell division, which may render a cancerous state to the normal tissue (14). Apoptosis is generally triggered by the induction of two signaling cascades, including the intrinsic and extrinsic pathways (15). A variety of cellular signaling pathways is implicated in the regulation of different components of apoptotic cell death (16). The activation of the Janus kinase/signal transducer and activator of the transcription (JAK2/STAT3) signaling pathway promotes the proliferation, migration, and invasion of cancer cells (17). It has been evidenced that the inhibition of the JAK2/STAT3 pathway potentiates apoptosis in cancer cells (18). In the present study, we aimed to explore the effects of corosolic acid on the viability and apoptosis of an invasive triple-negative breast cancer cell line, MDA-MB-231, and a non-invasive breast cancer cell line, MCF-7. Moreover, we investigated the possible mechanism of corosolic acid in inducing apoptosis in MDA-MB-231 cells. 

## Materials and Methods

***Cell culture and treatments***

Both MCF7 and MDA-MB-231 cell lines were purchased from National Center for Cell Science (Pune, India). The cells were then cultured and incubated in a humidified incubator at 37 ^°^C and 5% CO_2_. All the materials used for cell culture were purchased from Gibco, UK. Corosolic acid was purchased from Sigma-Aldrich (Germany). Both MCF7 and MDA-MB-231 cell lines were seeded at the density of 2×10^4^ cells/well into 96-well cell culture plates. Subsequently, the seeded cells were treated with different concentrations of corosolic acid (5, 10, 15, 20, 25, 30, 40, 50 μM) for 24, 48, and 72 hr.

***Cell viability***

After incubation of the cell lines with increasing concentrations of corosolic acid, the viability of the cells was quantified using Vybrant 3-(4,5-Dimethylthiazol-2-yl)-2,5-diphenyltetrazolium bromide (MTT) cell proliferation assay kit (Thermo Fischer Scientific, USA) according to the manufacturer’s instructions. Briefly, the cell lines were incubated with 5 mg/ml MTT solution for 4 hr to evaluate the influence of corosolic acid on their viability. Subsequently, the MTT solution was removed and 200 µl of dimethyl sulfoxide (DMSO) solution was added to each well. After 15 min, the quantity of formazan product was detected by measuring the absorbance at 570 and 630 nm using a plate reader spectrophotometer (PerkinElmer, USA).

***Real-time PCR***

The primary primer sequences were extracted from the NCBI database and the specific primers were designed by using Primer Express software v1.5 (Applied Biosystems). The total RNA content of both cell lines was extracted using an RNeasy mini kit (Qiagen, Korea). To synthesize the complementary DNA (cDNA) from 1 µg/ml of single-stranded RNA, the oligo-d (T) 15 primer (Roche Applied Sciences, Germany) and M-MLV reverse transcriptase were used. Real-time PCR analyses were carried out on cDNA samples by using SYBR-green master mix (Ampliqon, Denmark) on ABI PRISM 7900HT (Applied Biosystems, USA) under thermocycling conditions. Furthermore, melt curve analysis was carried out for each gene to prove the specificity of the primers and the lack of non-specific products. The expression levels of all genes were analyzed using the 2^-ΔΔCt^ method and the GAPDH transcript was used as the housekeeping gene. The primer sequences used in the present study are shown in Table 1.

***Flow cytometry***

The effect of corosolic acid on apoptosis was studied using a FITC/annexin-V-propidium iodide (PI) kit (apoptosis detection kit; R&D Systems) according to the manufacturer’s protocol. Briefly, MCF7 and MDA-MB-231 cells were treated with corosolic acid at concentrations equal to IC50 and IC25 for 48 hr. Then, the treated cells were centrifuged (1000 g) at room temperature (18-24 ^°^C) for 5 min, washed once with 5 ml of a phosphate-buffered saline (PBS), and resuspended in the binding buffer. Afterward, 5 µl of PI and 5 µl of FITC-annexin V were added to the cell suspension and incubated in the dark for 15 min at room temperature. Analysis of FITC-annexin V binding was carried out on a FAC scan flow cytometer (BD Biosciences) with an excitation wavelength of 488 nm and an emission wavelength of 350 nm. PI-negative/Annexin V-positive cells were presented as early apoptotic cells and the cells with positive PI and Annexin V were reported as late apoptotic cells. 

***Western blot analysis***

To measure the expression of proteins by Western blotting technique, both MCF7 and MDA-MB-231 cell lines were seeded in 6-well culture plates at the density of 2×10^5^ cells/well and allowed to adhere to the plates overnight. The following day the cultured cells were treated with IC50 and IC25 concentrations of corosolic acid for 48 hr. The cultured cells were subsequently harvested through centrifugation for 10 min, washed twice with PBS, and incubated in a RIPA lysis buffer (Thomas Scientific Inc., USA) to prepare the whole-cell lysates. The lysates were then centrifuged and the supernatants were utilized for Western blot analysis.

A bicinchoninic acid (BCA) assay kit (Thermo Fisher Scientific, UK) was used to estimate the total protein content of the samples. 40 µg of the total protein was loaded onto 10% SDS-PAGE and transferred onto a polyvinylidene fluoride (PVDF) membrane (Millipore, USA) through electroblotting. The membranes were blocked with 5% non-fat milk for 1 hr at room temperature. Subsequently, the membranes were exposed to Caspase-8, Caspase-9, Caspase-3, Bax, Bcl-2, STAT3, JAK2, Phosphorylated STAT3, Phosphorylated JAK2, and GAPDH primary antibodies overnight at 4 ^°^C. All membranes were then incubated with horseradish peroxidase (HRP)-conjugated secondary antibody in the dark for 1 hr at room temperature. All antibodies were purchased from Santa Cruz Biotechnology, Santa Cruz, CA, USA. Finally, the immunoblots were developed by using an enhanced chemiluminescent kit (SuperSignal, Thermo Fisher Scientific, UK) followed by exposure to autoradiography film.

***Measurement of the activity of caspase enzymes***

To measure the activity of Caspase-8, -3, and -9, both MCF7 and MDA-MB-231 cell lines were lysed in the lysis buffer (1% Triton X-100, 0.02 M Tris HCl pH 7.4, 1 mM EGTA, 250 mM sucrose, 1 mM EDTA, 150 mM NaCl, and 1 mM DTT) with vortexing for 30 min at 4 ^°^C. Subsequently, 200 μg of the total protein content of the cell lysate was mixed with the assay buffer (5% sucrose, 25 mM HEPES pH 7.5, 2 mM EDTA, 5 mM DTT, and 0.1% CHAPS) to a final volume of 100 μl. Then, 20 μl of each substrate (Caspase-9; Ac-LEHD-pNA, Sigma, USA), (Caspase-8; Z-IETD-pNA, Sigma, USA), (Caspase-3; Z-DEVDpNA, Sigma, USA) was added to the mixture. The reaction mixtures were incubated for 30 min at 37 ^°^C and the absorbance of the enzyme products was measured at 405 nm using an Epoch microplate spectrophotometer.

***Statistical analysis***

All experiments were repeated at least three times and the obtained data were expressed as mean±standard deviation (SD). The results were analyzed using the one-way analysis of variance (ANOVA) test followed by Duncan’s multiple range tests as the *post hoc* test. These statistical analyses were done using SPSS software version 19 (SPSS Inc., Chicago, IL, USA). The statistical difference between the data was considered significant at *P*<0.05.

## Results

***Effect of corosolic acid on the viability of MDA-MB-231 and MCF7 cell lines***

**Figure 1:**
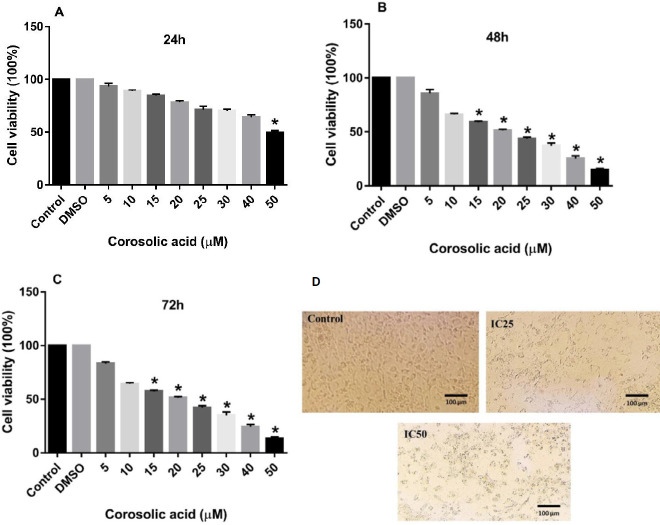
Anti-proliferative effect of corosolic acid on the MDA-MB-231 cell line. Different concentrations of corosolic acid (5, 10, 15, 20, 25, 30, 40, and 50 µM) were used to treat MDA-MB-231 cells for 24 (A), 48 (B), and 72 (C) hr. The morphology of the cells was shown in different groups (D). The MTT method was utilized to assess the viability of the cells. The data are expressed as mean±SD of three independent experiments. *P<0.05 was considered statistically significant

**Figure 2:**
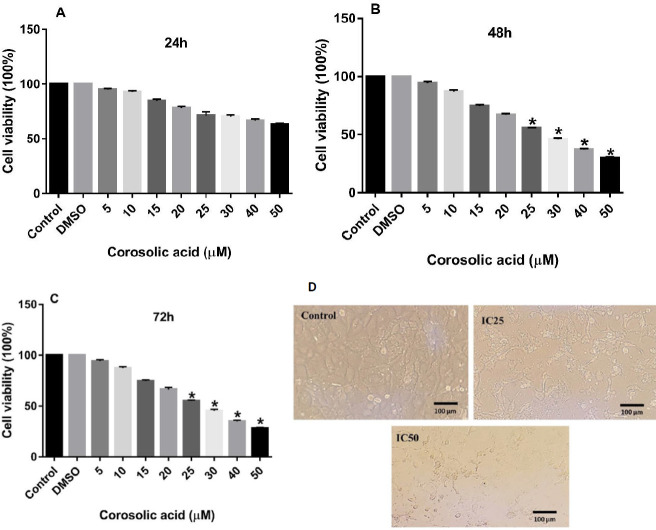
Anti-proliferative effects of corosolic acid on the MCF7 cell line. The cells were treated with different concentrations of corosolic acid (5, 10, 15, 20, 25, 30, 40, and 50 µM) for 24 (A), 48 (B), and 72 (C) hr. The morphology of the cells was shown in different groups (D). The cell viability was determined using an MTT assay. The data are expressed as mean±SD of three independent experiments. *P<0.05 was considered statistically significant

The results of the MTT assay revealed that treating both MDA-MB-231 and MCF7 cell lines with increasing concentrations of corosolic acid for 48 and 72 hr could mitigate the proliferation of these breast cancer cell lines in a dose-dependent manner compared with the untreated control cells. Corosolic acid treatment for 24 hr had no significant effects on either cancer cell line (Figures 1A and 2A). As presented in Figures 1B and C, corosolic acid significantly reduced the viability of the MDA-MB-231 cell line at the dose of 15 µM and more with a calculated IC50 value of 20.12 µM. However, corosolic acid hampered the cell viability in the MCF7 cell line at higher concentrations. The morphology of MDA-MB-231 cells treated with different concentrations of corosolic acid (0, 10, and 20 µM; 48 hr) was shown in Figure 1D. The figure indicates that IC25 and IC50 concentrations of corosolic acid lead to apoptotic death in the MDA-MD-231 cells with the characteristics of shrinkage and rounding of the cells. As shown in Figures 2B and C, treatment of MCF7 cells with corosolic acid at concentrations more than 25 µM significantly resulted in a considerable decrease in cell survival of these cancer cells in comparison with untreated controls. The IC50 value of the antiproliferative effect of corosolic acid on the MCF7 cell line for 48 hr was 28.50 µM. The morphology of MCF7 cells treated with different concentrations of corosolic acid (0, 14, and 28 µM; 48 hr) was shown in Figure 2D. Cell shrinkage and rounding were also observed but in less extent. This can suggest the involvement of a non-apoptotic mechanism in corosolic acid-induced cell death of MCF-7 cells.

***Effects of*** ***corosolic acid on apoptosis induction in MDA-MB-231 and MCF7 cell lines***

**Figure 3:**
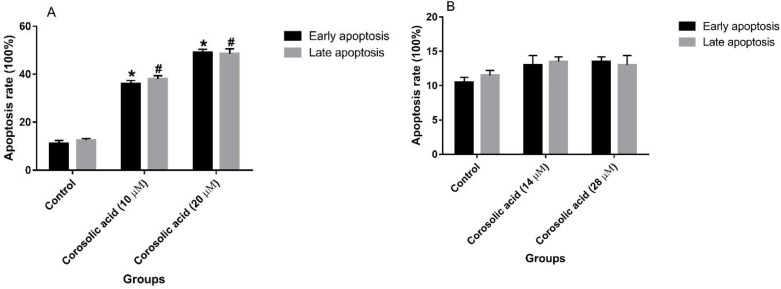
(A) Apoptosis-inducing effect of corosolic acid on MDA-MB-231 cell line at the doses of 10 (IC25) and 20 (IC50) µM for 48 hr. (B) Apoptosis-inducing effect of corosolic acid on MCF7 cell line at the doses of 14 (IC25) and 28 (IC50) µM for 48 hr. The data represented as means±SD for the three independent experiments (data were considered significant at *P<0.05 and ^#^*P<*0.05, compared with the controls)

To evaluate the apoptosis-inducing activity of corosolic acid on MDA-MB-231 and MCF7 cell lines, the cells were treated with IC25 and IC50 concentrations of this natural compound. In the MDA-MB-231 cell line, a marked increase in the late and early stages of apoptosis was observed after treatment of the cell line with 10 μM (IC25) and 20 μM (IC50) of corosolic acid for 48 hr (Figure 3A). Corosolic acid at 20 μM resulted in a non-significant elevation in the number of apoptotic cells in comparison with 10 μM corosolic acid. Treatment of the MCF7 cell line with 14 μM (IC25) and 28 μM (IC50) doses of corosolic acid did not change either late- or early-stage apoptotic cell populations as compared with the untreated control cells (Figure 3B).

***Effects of corosolic acid on caspase enzyme activity in MDA-MB-231 and MCF7 cell lines***

**Figure 4:**
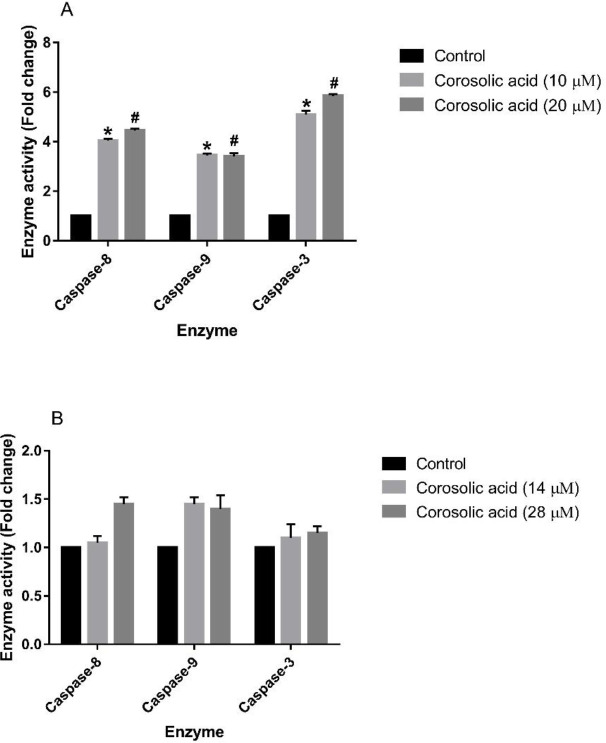
(A) Effect of corosolic acid treatment at the doses of 10 (IC25) and 20 (IC50) µM for 48 hr on the activity of Caspase-8, -9, and -3 in the MDA-MB-231 cell line. (B) Effect of corosolic acid treatment (14 (IC25) and 28 (IC50) µM for 48 hr) on the activity of Caspase-8, -9, and -3 in the MCF7 cell line (#*P<*0.05 and *P<0.05, compared with the corresponding controls). The experiments were performed in triplicate. The results are representative of means±SD. #*P<*0.05 and *P<0.05 were considered statistically significant

After corosolic acid treatment, the activity of three key caspase enzymes, Caspase-8, -3, and -9 was measured in MDA-MB-231 and MCF7 cell lines to evaluate the activation and function of these enzymes in the presence of this compound. As depicted in Figure 4A, corosolic acid significantly elevated the enzyme activity of Caspase-8, -3, and -9 in the MDA-MB-231 cell line in comparison with the untreated control cells. Treating this cell line with both 10 µM and 20 µM corosolic acid showed its enhancing effects on the function of all three caspase enzymes. Nonetheless, the treatment of MCF7 cells with 14 µM and 28 µM of corosolic acid had no significant effect on the activity of the mentioned caspase enzymes as compared with the control cells (Figure 4B). 

***Effects of corosolic acid on expression of apoptosis-related factors*** ***in MDA-MB-231 cell line***

**Figure 5:**
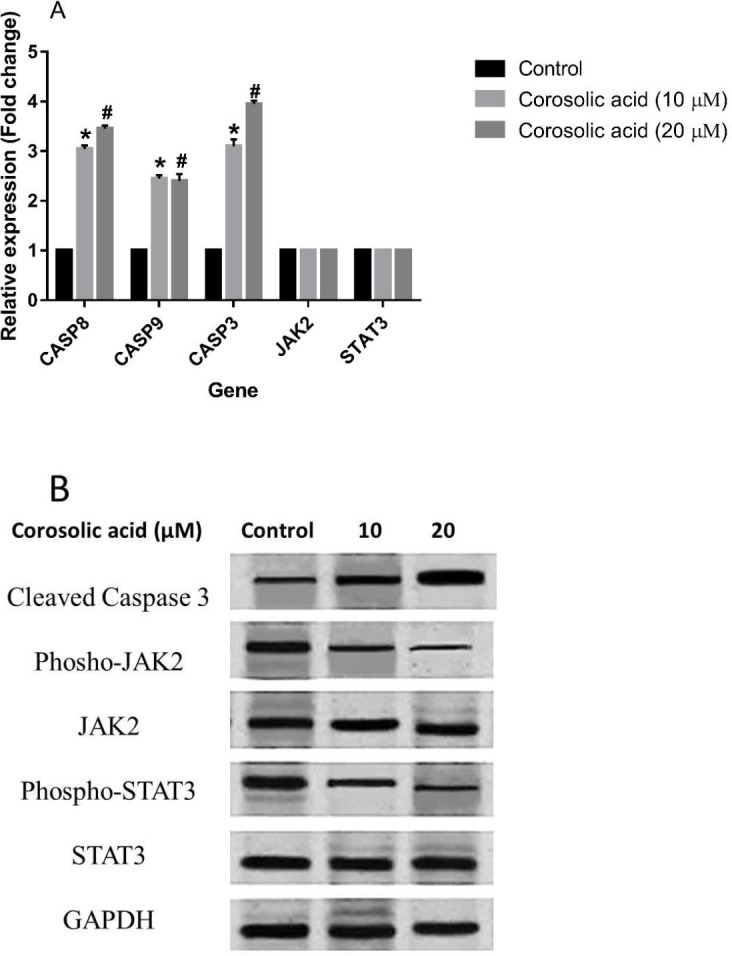
(A) Effect of corosolic acid treatment on the expression of CASP8, CASP9, CASP3, JAK2, and STAT3 genes in the MDA-MB-231 cell line. (B) Effect of corosolic acid treatment on the expression and phosphorylated and unphosphorylated forms of JAK2 and STAT3 in the MDA-MB-231 cell line. #*P<*0.05 and *P<0.05 were considered statistically significant

To assess the possible effect of corosolic acid on key factors involved in the apoptosis of MDA-MB-231 cells, the expression levels of some apoptosis-related genes and proteins were measured. The results of quantitative real time-PCR analyses provided evidence to show that corosolic acid, at concentrations equal to IC25 and IC50 for 48 hr treatment, considerably elevated the transcript levels of CASP-8, -9, and -3 in the MDA-MB-231 cell line in comparison with untreated control cells. However, it had no significant effect on the expression levels of JAK2 and STAT3 genes compared with the controls (Figure 5A). The results of western blotting showed that the activated form of Caspase-3 protein was enhanced after treating the cells with the mentioned concentrations of corosolic acid, indicating the apoptosis-inducing activity of this natural compound in the MDA-MB-231 cell line. Additionally, the protein expression results showed that corosolic acid could hinder the activation of both JAK2 and STAT3 proteins within these cancer cells by inhibiting the phosphorylation and activation of these proteins. However, it had no remarkable effect on the expression levels of JAK2 and STAT3 proteins (Figure 5B).

***Effects of corosolic acid on expression of apoptosis-related factors*** ***in MCF7 cell line***

**Figure 6:**
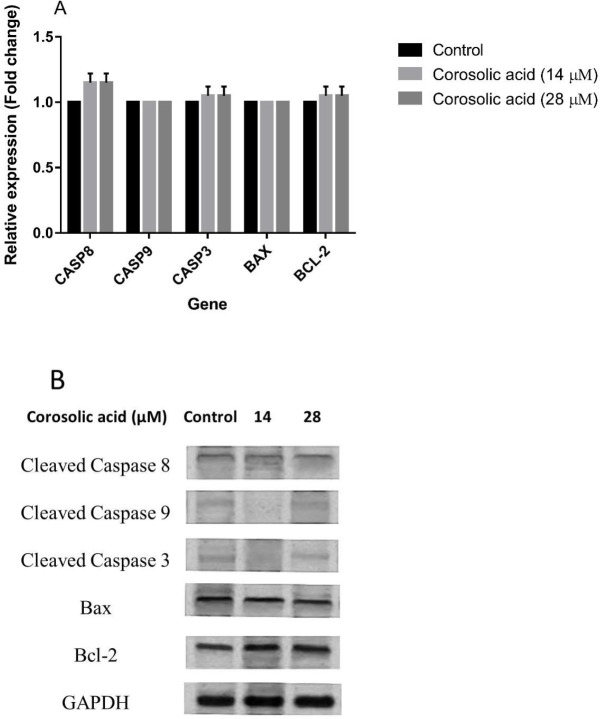
(A) Effect of corosolic acid treatment on the mRNA expression of CASP8, CASP9, CASP3, BAX, and BCL-2 genes in the MCF7 cell line. (B) Effect of corosolic acid on the protein expression of Caspase-8, -9, -3, Bax, and Bcl-2 in the MCF7 cell line. The data represented as means±SD for the three separate experiments. #*P<*0.05 and *P<0.05 were considered statistically significant

To unravel the potential effect of corosolic acid on apoptosis markers in MCF7 cells, the gene and protein expression of these markers were quantified using real time-PCR and western blotting. The results of gene expression analysis uncovered that corosolic acid treatment of MCF7 cells with IC25 (14 µM) and IC50 (28 µM) concentrations of this agent for 48 hr did not alter the transcript expression of CASP-8, -9, and -3 as well as BAX and BCL-2 as compared with the untreated control cell line (Figure 6A). The results of western blotting also affirmed that corosolic acid had no remarkable activity in the augmentation of the anti-apoptotic Bal-2 marker, activation of Caspase-8, Caspase-9, and Caspase-3 as well as the suppression of pro-apoptotic marker Bax in MCF7 cells (Figure 6B).

## Discussion

As described before, TNBC is the most aggressive type of breast cancer and develops resistance to treatment methods due to the absence of ER, PR, and HER-2 receptors (3). Although some therapies are available, therapy resistance is the major problem in the treatment of TNBC (19). However, hormone-positive subtypes of breast cancer may benefit from hormone therapy methods and have better survival rates (20). Consequently, new research should focus on the discovery of novel and effective therapeutic modalities for TNBC. Apoptosis is an important programmed cell death process that can be an effective and ideal target for cancer therapy (21). Therefore, the present study was designed to evaluate the potential effect of corosolic acid on the induction of apoptosis in an invasive, MDA-MB-231, and a non-invasive, MCF7, subtype of breast cancer.

In the first step, MDA-MB-231 and MCF7 cell lines were treated with increasing concentrations of corosolic acid for 24, 48, and 72 hr. Corosolic acid treatment of both MDA-MB-231 and MCF7 cell lines led to a significant decrease in the viability of the cells in a dose- and time-dependent manner. Of note, this triterpenoid hindered the MDA-MB-231 cell proliferation rate at lower concentrations (IC50=20.12 µM, 48 hr) than those in the MCF7 cell line (IC50=28.50 µM, 48 hr). This suggests that the TNBC cell line, MDA-MB-231, is more sensitive to corosolic acid than the PR- and ER-positive MCF7 cell line. Additionally, it can be implied that the anti-breast cancer effect of corosolic acid is not necessarily receptor-dependent. 

In the second step, we hypothesized that the antiproliferative activity of corosolic acid may arise from its effect on the induction of apoptosis in both cell lines. Hence, the cell lines were treated with IC50 and IC25 concentrations of corosolic acid. The results clarified that corosolic acid considerably augmented both early and late apoptotic cell populations of the MDA-MB-231 cell line, however, it showed no remarkable pro-apoptotic activity against MCF7 cells. To affirm the role of the key caspase enzymes in this effect, the cell lines were exposed to IC50 and IC25 concentrations of corosolic acid, and then the activity of Caspase-8, -9, and -3 was measured. The obtained findings confirmed the elevated activity of all three caspases in the MDA-MB-231 cell line due to corosolic acid treatment. However, the compound did not affect the activity of the enzymes in the MCF7 cell line. The process of apoptosis functions through two main pathways that include mitochondria-dependent (intrinsic) and death receptor-mediated (extrinsic) pathways. The former pathway initiates apoptosis via activating Caspase-9 and the latter induces apoptosis by stimulating Caspase-8. These activated caspases trigger the activation of Caspase-3, as the executioner in apoptosis (22). Accordingly, it seems that corosolic acid induces apoptosis in MDA-MB-231 cells by triggering both pathways of apoptosis, indicating a potential mechanism of action for the anti-TNBC activity of corosolic acid. 

In the third step, the effect of corosolic acid treatment on the expression levels of some apoptosis-related genes and proteins was quantified in MDA-MB-231 and MCF7 cell lines. The results of real-time PCR and western blotting methods evidenced that corosolic acid had no marked effect on the expression levels of Caspase-8, -9, -3, Bax, and Bcl-2 at either mRNA or protein levels. However, treating the MDA-MB-231 cell line with IC50 and IC25 concentrations of corosolic acid significantly amplified the levels of Caspase-8, -9, and -3 in comparison with untreated control cells. 

To the best of our knowledge, only three studies in the literature investigated the effect of corosolic acid on breast cancer. For example, the results of an investigation showed that corosolic acid inhibited the proliferation of MCF7 cells by blocking factor-kappa B (NF-κB) activity. However, they did not study the apoptosis-inducing activity of corosolic acid in this cell line (23). Son *et al.* identified that corosolic acid decreased the proliferation and increased apoptosis of MDA-MB-231 cells in a dose-dependent manner. They revealed that the apoptosis-inducing effect of corosolic acid on MDA-MB-231 cells was mediated by a mechanism that involves reactive oxygen species (ROS) (24). However, they did not describe which pathway of apoptosis is involved in the apoptosis-inducing effect of corosolic acid on this cell line. Jiang *et al*. demonstrated that corosolic acid displayed anticancer capacity against MDA-MB-231 cells by inhibiting cell proliferation and inducing apoptosis through a mechanism that regulates the p38/MAPK signaling pathway (25). They measured Caspase-3 expression and Bax/Bcl-2 ratio and concluded that corosolic acid triggers apoptosis in MDA-MB-231 cells via the intrinsic pathway. However, they did not determine the levels or activity of Caspase-8 and -9, which are pivotal markers of extrinsic and intrinsic apoptosis, respectively. On the contrary, the present study estimated the activity and levels of all three key caspase enzymes and then concluded that corosolic acid induces both pathways of apoptosis in MDA-MB-231 cells. A study proved that treating osteosarcoma MG-63 cells with corosolic acid led to the activation of caspase-8, -9, and -3, suggesting the stimulation of both the intrinsic and extrinsic pathways in these cancer cells (26). Sung and colleagues reported that corosolic acid promoted apoptosis in HCT116 human colon cancer cells by acting on both pathways of apoptosis (10). However, some studies, which have evaluated the effect of corosolic acid on cervix adenocarcinoma, lung adenocarcinoma, and NCI-N87 gastric cancer cell lines, showed that this compound triggers apoptosis in these cells by activating the intrinsic pathway (27-29). It seems that the controversy between the results of these studies arises from the difference between the types of cancer cells. Therefore, corosolic acid may use different mechanisms in various types of cancer.

An increasing body of evidence in the literature shows that targeting the JAK/STAT signaling pathway in TNBC cell lines is a novel and efficient approach to stimulating apoptotic cell death in these cancer cells (30-32). Consequently, the expression levels of JAK2 and STAT3 as well as their phosphorylated forms were measured in the MDA-MB-231 cell line. The obtained findings evidenced that corosolic acid did not affect the expression of unphosphorylated forms of JAK2 and STAT3. However, it diminished the levels of their phosphorylated forms as compared with the control cells. These data are in line with the previous studies. Fujiwara *et al.* demonstrated that corosolic acid suppressed the proliferation of glioblastoma cells and tumor-associated macrophages by blocking STAT3 and NF-κB in these cells (33). It has been reported that the inhibition of the JAK/STAT pathway in cancer cells could lead to the induction of apoptosis in these cells and may result in tumor suppression (34). Corosolic acid, in the present investigation, played a role as an inhibitor of the JAK/STAT signaling pathway. Thus, inhibition of the JAK/STAT pathway could be another potential mechanism by which corosolic acid triggers apoptosis in the MDA-MB-231 cells.

**Table 1:** Primer sequences used in this study of gene expression analysis

Gene name	Forward primer	Reverse primer
CASP8	5'-GTGGGGTAATGACAATCTCGG-3′	5'-TCAAAGGTCGTGGTCAAAGC-3′
CASP9	5'- GCAGGCTCTGGATCTCGGC-3′	5'-GCTGCTTGCCTGTTAGTTCGC-3′
CASP3	5′ -CTCGGTCTGGTACAGATGTCGATG-3′	5’-GGTTAACCCGGGTAAGAATGTGCA-3′
STAT3	5'-GCTTCCTGCAAGAGTCGAAT-3′	5'-ATTGGCTTCTCAAGATACCTG-3′
JAK2	5'-GATGAGAATAGCCAAAGAAAACG-3′	5'-TTGCTGAATAAATCTGCGAAAT-3′
BAX	5'-CCCTTTTGCTTCAGGGTTTCAT-3′	5'-ACTCGCTCAGCTTCTTGGTG-3′
BCL-2	5'-CTGTGGATGACTGAGTACCT-3′	5'-GCCAGGAGAAATCAAACAGAG-3′
GAPDH	5'-ACCCACTCCTCCACCTTTGA-3′	5'-CT GTTGCTGTAGCCAAATTCGT-3′

## Conclusion

Natural products that target both pathways of apoptosis are the most competent anti-cancer drugs because they prevent drug resistance that arises from the cross-talks between the signaling pathways in the cancer cells (35). Therefore, the drugs that simultaneously target more than one pathway are more effective in cancer suppression. Our data also evidenced that corosolic triggers both pathways of apoptosis in the MDA-MB-231 cell line and this makes it an efficient candidate for the treatment of TNBC. The findings of this study also discovered that corosolic acid-induced apoptosis in MDA-MB-231 cells by suppressing the activation of the JAK/STAT pathway. Therefore, the present data suggested two mechanisms of action for the apoptosis-inducing activity of corosolic acid against the TNBC MDA-MB-231 cell line. Moreover, further experiments showed that corosolic acid reduced the viability of MCF7 cells, but had no apoptotic effect on this PR- and ER-positive cell line, suggesting the involvement of a non-apoptotic mechanism in anti-proliferative activity of corosolic acid against MCF7 cells. These findings also suggest that the anti-breast cancer effects of corosolic acid are mediated in a receptor-independent manner. Taken together, corosolic acid may be considered an anti-proliferative agent for different types of breast cancer with pro-apoptotic effects on TNBC. 

Although the present study has valuable data and offers beneficial contributions to current literature, we note some limitations of our research and suggest some extra evaluations for future studies. As indicated in the title of the manuscript, the present study is an *in vitro* investigation of the effects of corosolic acid on breast cancer cell lines. Thus, future works should focus on the development of *in vivo* models of breast cancer using both cancer cell lines. Then, the present factors can be evaluated in those animal models to establish the present results.

## Authors’ Contributions

KH, ATJ, and SZ designed the experiments and methodology. SAJ, OZK, RSZ, QAQ, and YFM performed the experiments and collected the data. SHA, CMW, YABJ, and MSM were involved in data analysis and manuscript writing. KH edited the manuscript. 

## Conflicts of Interest

The authors have no conflicts of interest to declare.
